# Impact of membrane lung surface area and blood flow on extracorporeal CO_2_ removal during severe respiratory acidosis

**DOI:** 10.1186/s40635-017-0147-0

**Published:** 2017-08-01

**Authors:** Christian Karagiannidis, Stephan Strassmann, Daniel Brodie, Philine Ritter, Anders Larsson, Ralf Borchardt, Wolfram Windisch

**Affiliations:** 1 Department of Pneumology and Critical Care Medicine, Cologne-Merheim Hospital, ARDS and ECMO Centre, Kliniken der Stadt Köln gGmbH, Witten/Herdecke University Hospital, Ostmerheimer Strasse 200, D-51109 Cologne, Germany; 2 Division of Pulmonary, Allergy and Critical Care, Columbia University College of Physicians and Surgeons/New York-Presbyterian Hospital, New York City, NY USA; 3 Enmodes GmbH, Aachen, Germany; 4 Hedenstierna Laboratory, Anesthesiology and Intensive Care, Department of Surgical Sciences, Uppsala University, Uppsala, Sweden

**Keywords:** ECMO, ECCO_2_R, Severe COPD, Exacerbation, Asthma, Renal replacement therapy, Low-flow

## Abstract

**Background:**

Veno-venous extracorporeal CO_2_ removal (vv-ECCO_2_R) is increasingly being used in the setting of acute respiratory failure. Blood flow rates through the device range from 200 ml/min to more than 1500 ml/min, and the membrane surface areas range from 0.35 to 1.3 m^2^. The present study in an animal model with similar CO_2_ production as an adult patient was aimed at determining the optimal membrane lung surface area and technical requirements for successful vv-ECCO_2_R.

**Methods:**

Four different membrane lungs, with varying lung surface areas of 0.4, 0.8, 1.0, and 1.3m^2^ were used to perform vv-ECCO_2_R in seven anesthetized, mechanically ventilated, pigs with experimentally induced severe respiratory acidosis (pH 7.0–7.1) using a 20Fr double-lumen catheter with a sweep gas flow rate of 8 L/min. During each experiment, the blood flow was increased stepwise from 250 to 1000 ml/min.

**Results:**

Amelioration of severe respiratory acidosis was only feasible when blood flow rates from 750 to 1000 ml/min were used with a membrane lung surface area of at least 0.8 m^2^. Maximal CO_2_ elimination was 150.8 ml/min, with pH increasing from 7.01 to 7.30 (blood flow 1000 ml/min; membrane lung 1.3 m^2^). The membrane lung with a surface of 0.4 m^2^ allowed a maximum CO_2_ elimination rate of 71.7 mL/min, which did not result in the normalization of pH, even with a blood flow rate of 1000 ml/min. Also of note, an increase of the surface area above 1.0 m^2^ did not result in substantially higher CO_2_ elimination rates. The pressure drop across the oxygenator was considerably lower (<10 mmHg) in the largest membrane lung, whereas the smallest revealed a pressure drop of more than 50 mmHg with 1000 ml blood flow/min.

**Conclusions:**

In this porcine model, vv-ECCO_2_R was most effective when using blood flow rates ranging between 750 and 1000 ml/min, with a membrane lung surface of at least 0.8 m^2^. In contrast, low blood flow rates (250–500 ml/min) were not sufficient to completely correct severe respiratory acidosis, irrespective of the surface area of the membrane lung being used. The converse was also true, low surface membrane lungs (0.4 m^2^) were not capable of completely correcting severe respiratory acidosis across the range of blood flows used in this study.

**Electronic supplementary material:**

The online version of this article (doi:10.1186/s40635-017-0147-0) contains supplementary material, which is available to authorized users.

## Background

Extracorporeal CO_2_ removal (ECCO_2_R) is increasingly recognized as a potentially valuable therapeutic option for patients with acute respiratory failure. ECCO_2_R has been used in a variety of settings, and may be used to correct severe respiratory acidosis [1]. ECCO_2_R has generated widespread interest for its potential application in acute exacerbations of chronic obstructive pulmonary disease (COPD), both in avoiding endotracheal intubation in patients at risk of failing non-invasive ventilation (NIV) [2], as well as decreasing the duration of invasive mechanical ventilation following intubation [3–5]. ECCO_2_R may also facilitate highly protective mechanical ventilation using volumes and pressures below the currently accepted standard of care with the goal of further reducing ventilator-induced lung injury [6–9].

From a technical perspective, different approaches to ECCO_2_R have been developed. Pumpless arterio-venous extracorporeal CO_2_ removal (av-ECCO_2_R), using the natural pressure gradient between the peripheral arterial and venous system to drive blood across the membrane oxygenator, as well as pump-driven veno-venous ECCO_2_R, are both currently used in clinical practice [7, 10]. However, epidemiological data has shown that the number of patients receiving av-ECCO_2_R is steadily decreasing, while the number of those receiving vv-ECCO_2_R, is increasing over time [11].

Historically, vv-ECCO_2_R has manly arisen from two areas of organ replacement. In the first instance, low-flow vv-ECCO_2_R is based on systems used for hemodialysis [8, 9, 12–15]. Second, high-flow extracorporeal membrane oxygenation, originally used for the treatment of severe acute respiratory distress syndrome (ARDS), has also been used for ECCO_2_R, even though the blood flow for CO_2_ removal alone are considerably less than those needed to provide oxygenation to a profoundly hypoxemic ARDS patient [3, 16, 17]. Third, there are only very few specifically designed systems with the primary aim of CO_2_ removal [18]. However, blood flow rates used for vv-ECCO_2_R vary considerably among different studies with blood flow rates ranging between 200 and 1800 mL/min [19].

Recently, we have shown in a porcine model that ECCO_2_R was most effective in correcting severe respiratory acidosis when using blood flow rates ranging between 750 and 1000 mL/min and a membrane lung with an area of approximately 1.0 m^2^, while lower blood flow rates were not sufficient to correct severe respiratory acidosis [1]. However, different systems are currently used for vv-ECCO_2_R in clinical practice also utilizing blood flows between 250 and more than 1000 ml/min with different membrane lungs. While the configuration of the membrane lung likely impacts the capability to remove CO_2_, its surface area is believed to be the key factor for sufficient CO_2_ removal, and, in this regard, there is a broad heterogeneity in clinical practice with the surface areas of oxygenators ranging from 0.35 to 1.3 m^2^, respectively [2, 3, 8, 9, 16, 17, 19, 20].

Theoretically, small oxygenators may be preferable as the blood velocity is suggested to be higher in these devices compared with larger oxygenators, and the results of previous computational fluid dynamic studies have demonstrated that low velocity regions qualitatively matched regions with a high incidence of thrombotic deposition [21, 22]. In this regard, lower blood flow rates also contribute to a low blood velocity within the membrane lung increasing the likelihood of clotting. This, in turn, would require more aggressive anticoagulation with a consequent increased bleeding risk. However, a high pressure drop over the membrane lung, which increases with decreasing membrane lung size, is associated with increased hemolysis [23, 24].

Interestingly, a recent clinical study showed that vv-ECCO_2_R was capable of preventing endotracheal intubation in COPD patients at risk of failing NIV, however, the rate of clotting was notably high, and this may be attributed to the low blood flow of 255 ± 78 mL/min and the large size of the membrane lungs used: 1.35 m^2^ [2]. In addition, smaller sized membrane lungs may be advantageous when applied for longer durations, as in those using ECCO_2_R for bridging to lung transplantation, or when serving as long-term paracorporeal lung support.

On the other hand, larger membrane lungs are thought to be more efficient. In addition, a larger surface area is usually associated with less pressure drop across the oxygenator resulting in better blood compatibility. Of note, there is no study, which has systematically investigated the impact of different sizes of membrane lungs used for vv-ECCO_2_R on the capability of removing elevated PaCO_2_ levels and on flow characteristics within the oxygenator. Therefore, the present study, using a porcine model with similar CO_2_ production as an adult resting human, was aimed at systematically investigating the impact of different sizes of membrane lungs on their efficiency and on flow characteristics in the setting of varying blood flow rates within the range most typically used for vv-ECCO_2_R.

## Methods

### Extracorporeal CO_2_ removal (ECCO_2_R) techniques

For the vv-ECCO_2_R system, four different membrane lungs (Maquet Cardiopulmonary Care, Rastatt, Germany) based on the Rotaflow® platform were used. The membrane lungs consisted on a polymethylpentene membrane with surface areas of 0.4, 0.8, 1.0, and 1.3m^2^. However, the membrane lung with 1.0 m^2^ is the only one lacking heat exchanging fibers. All four membrane lungs have a comparable rhomboid design. The systems were primed with normal saline solution. Heparin (5000 IE) was added to the running system and bolus application of 5000 IE every 2–3 h was used during the running of the systems to avoid clotting.

For venous access, a 20Fr Bicaval Avalon ELITE Dual Lumen Cannula® (Maquet Cardiopulmonary Care, Rastatt, Germany) was inserted into the right jugular vein. Correct placement of the cannula was confirmed by echocardiography. Furthermore, the partial pressure of carbon dioxide was measured regularly pre- and post-membrane lung, i.e., directly before and after passing through the membrane lung. Lower values of PCO_2_ post-membrane lung compared to the partial pressure of arterial carbon dioxide (PaCO_2_) indicated a low recirculation rate and an optimized cannula position (Additional file 1: Figure S1). For all experiments, the sweep gas flow was constantly set to 8 L/min with a fraction of delivered oxygen at 1.0. This was based on previous findings showing that sweep gas flow rates higher than 8 L/min did not result in a substantial increase in CO_2_ removal under these defined conditions [1, 25].

### Animal model

The study was approved by the Animal Research Committee of Uppsala University in Sweden (ethical approval number: C77/16). Pigs (body weight = 44.6 ± 3.8 kg) were anesthetized with IV ketamine 25 to 50 mg/kg/h, midazolam 90 to 180 μg/kg/h, fentanyl 3 to 6 μg/kg/h, and rocuronium 2.5 to 5.0 mg/kg/h was added when adequate anesthesia was ascertained by lack of response of painful stimulation between the front hooves. The trachea was intubated with a cuffed endotracheal tube (inner diameter, 7 mm). The pigs were ventilated with a Servo-i ventilator (Maquet Critical Care, Solna, Sweden). Body temperature was kept at 38 °C throughout the study period by use of a warming blanket. Arterial blood was sampled from the left carotid artery. The estimated carbon dioxide (CO_2_) production is about 200–280 ml/min in pigs [26, 27], which is comparable to an adult human.

### Study design

Vv-ECCO_2_R was performed in seven pigs following endotracheal intubation, mechanical ventilation and induction of respiratory acidosis by increased dead space ventilation. In detail, anatomical dead space was increased by adding a further tube between the endotracheal tube and the “Y” piece of the ventilator circuit. The length of the additional tube was titrated until respiratory acidosis was induced with a target pH value ranging between 7.0 and 7.1. Pigs were ventilated in a volume-controlled mode with a tidal volume of 220–250 ml, a positive end-expiratory pressure of 5 cm H_2_O and a breathing frequency of 14–16/min, aiming at a target pH value between 7.0 and 7.1. The dead space fraction was subsequently maintained for the entire duration of the experimental period.

Experiments were performed in each pig in a standardized fashion. Each pig received all four membrane lungs during the day. Equal conditions were used across all experiments (lasting at least 30 min), with each experiment starting at the pre-determined acidotic conditions. Blood flow rates were increased in a stepwise fashion. Each step lasted 30 min to achieve equilibrium conditions, with all measurements taken at the end of this 30-min period.

### CO_2_ and blood gas measurement

Blood gas analysis was performed with an ABL 800, Radiometer, (Copenhagen, Denmark) with separate measurement for hemoglobin. Extracorporeal CO_2_ removal was calculated as follows:

1. CO_2_-transfer membrane lung [ml/min] = (ctCO_2_ (blood) before membrane lung–ctCO_2_ (blood) after membrane lung)× blood flow [L/min]× 10
2. ctCO_2_ (blood) = 9.286× 10^−3^× pCO_2_× ctHb$\times \left\lfloor 1+{10}^{\Big({pH}_{Ery}-{pK}_{Ery}}\right\rfloor$
  + ctCO_2_ (plasma)$$ \times \left(1-\frac{\mathrm{ctHb}}{21.0}\right) $$
3. pH_Ery_ = 7.19 + 0.77× (pH −7.40) + 0.035× (1 − sO_2_)
  pK_Ery_ = 6.125−log$$ \left\lfloor 1+{10}^{\left({pH}_{Ery}-7.48-0.06\times \mathrm{s}{\mathrm{O}}_2\right)}\right\rfloor $$
4. ctCO_2_ (plasma) = 0.23× pCO_2_ + cHCO_3_^−^ (plasma)
5. cHCO3^−^ (plasma) = 0.23× pCO_2_× 10^(pH−pK^_P_^)^

(ctCO_2_: carbon dioxide content; HCO3-: bicarbonate; pH_Ery_: erythrocyte pH value; ctHb: concentration of hemoglobin in the blood).

### Statistics

For statistical analysis, GraphPad Prism 7 for Macintosh computer (La Jolla, CA 92037, USA) was used. Data were tested for normality using the Kolmogorov-Smirnov test with a cut-off *p* value of <0.05. Normally distributed data are expressed as mean and standard deviation.

## Results

**Fig. 1:**
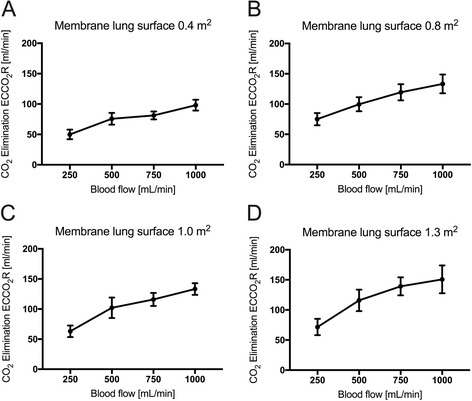
Extracorporeal elimination of carbon dioxide (CO_2_) depending on blood flow. Membrane lung surface ranges from 0.4 m^2^ (**a**), 0.8 m^2^ (**b**), 1.0 m^2^ (**c**) to 1.3 m2 (**d**) with a sweep gas flow of 8 L O_2_/min. Blood flow was titrated from 250 to 1000 ml/min. Each data point represents the mean and standard deviation of seven pigs

**Fig. 2:**
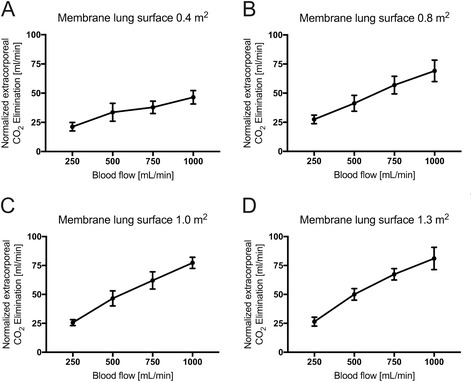
Normalized extracorporeal elimination of carbon dioxide (CO_2_) depending on blood flow. Normalized CO_2_ removal was calculated by normalizing the partial pressure of carbon dioxide before the membrane lung to 45 mmHg. Membrane lung surface ranges from 0.4 m^2^ (**a**), 0.8 m^2^ (**b**), 1.0 m^2^ (**c**) to 1.3 m2 (**d**) with a sweep gas flow of 8 L O_2_/min. Blood flow was titrated from 250 to 1000 ml/min. Each *data point* represents the mean and standard deviation of seven pigs

**Fig. 3:**
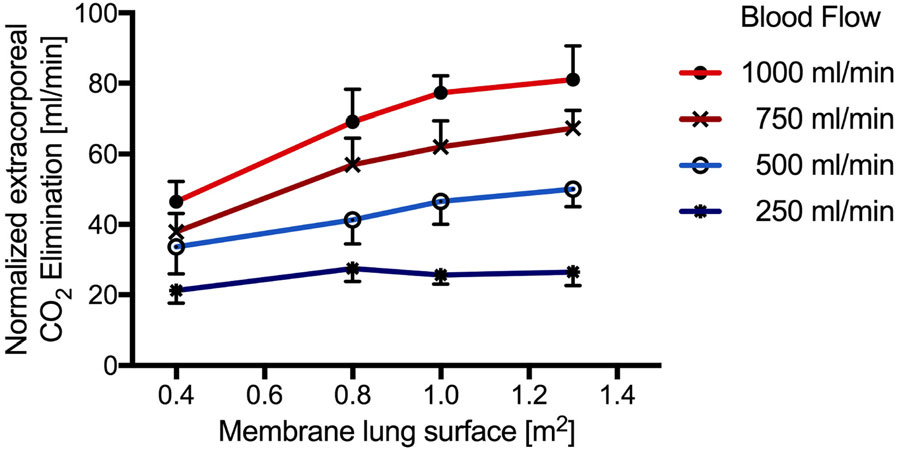
Normalized extracorporeal elimination of carbon dioxide (CO_2_) depending on membrane lung surface. Normalized CO_2_ removal was calculated by normalizing the partial pressure of carbon dioxide before the membrane lung to 45 mmHg. The normalized extracorporeal CO_2_ elimination was plotted against membrane lung surface. Blood flow was titrated from 250 to 1000 ml/min. Each *data point* represents the mean and standard deviation of seven pigs

**Fig. 4:**
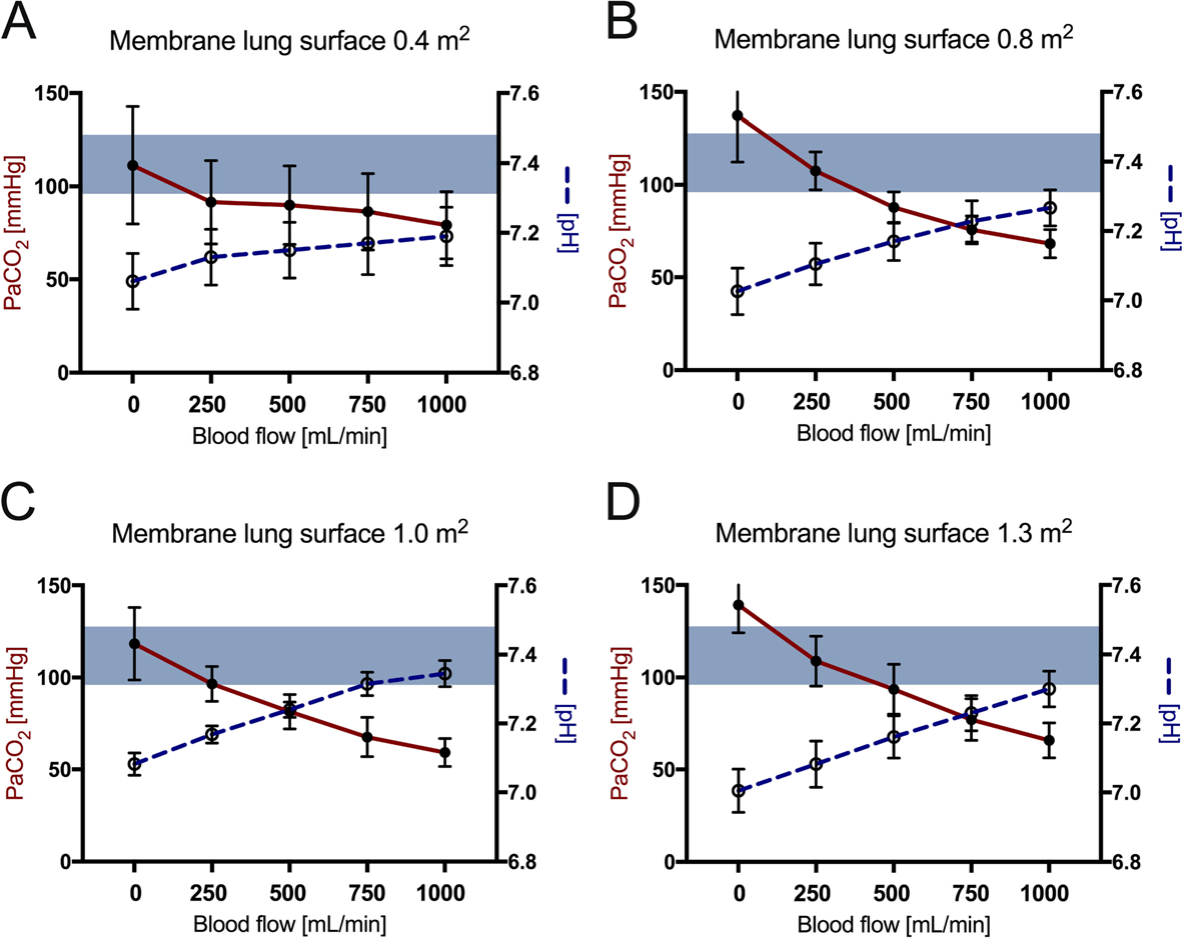
Partial pressure of arterial carbon dioxide (PaCO_2_/*red line*) and corresponding pH value (*blue dotted line*) depending on blood flow. Membrane lung surface ranges from 0.4 m^2^ (**a**), 0.8 m^2^ (**b**), 1.0 m^2^ (**c**) to 1.3 m2 (**d**) with a sweep gas flow of 8 L O_2_/min. Blood flow was titrated from 250 to 1000 ml/min. Each *data point* represents the mean and standard deviation of seven pigs

For better comparison of the efficiency of the membrane lung (Fig. 1), normalized CO_2_ removal was calculated by normalizing the partial pressure of carbon dioxide before the membrane lung to 45 mmHg as described before [28] (Figs. 2 and 3) under each condition. Normalization is important since PaCO_2_ and PvCO_2_ levels were, under some conditions, even above 130 mmHg with a certain variety (Additional file 1: Figure S1 and Additional file 2: Figure S3). ECCO_2_R was most effective across all membrane sizes with the highest blood flow rate of 1000 ml/min (Figs. 1, 2, 3, and 4, Tables 1, 2, and 3). While all membrane lungs with a surface of ≥0.8 m^2^ revealed a linear and clear increase in CO_2_ removal with increasing blood flow rates (Figs. 1, 2, and 3), the smallest membrane lung showed only a small increase and nearly steady state condition with blood flow rates of more than 500 ml/min (Figs. 1, 2, and 3). Compared to the 0.8 m^2^ membrane lung, at a blood flow rate of 1000 ml/min, a surface area of 1.0 m^2^ (25% more surface area) allowed an 11.8% increase in efficiency, and the 1.3 m^2^ membrane lung, with 62.5% more surface area, only a 17.3% increase (Table 3). Thus, partial pressure of CO_2_ in arterial blood (PaCO_2_) progressively decreased with increasing blood flow rates corresponding to extracorporeal CO_2_ removal rates (Fig. 4, Table 2). However, severe respiratory acidosis could only be corrected towards a pH value of >7.30 with blood flow rates of 1000 ml/min (Fig. 4, Table 1), except the smallest membrane lung, which was unable to correct the severe acidosis even with a blood flow of 1000 ml/min (Fig. 4, Table 1). All three membrane lungs with a surface of ≥0.8 m^2^ could reduce the initial PaCO_2_ by 50–53% with a blood flow rate of 1000 ml/min (Fig. 4). Similarly, pH values progressively increased in proportion with blood flow (Fig. 4, Table 1). Of note, fully correcting the initial severe respiratory acidosis (pH 7.01–7.08) was only possible with blood flow rates of 1000 ml/min, and only when using membrane lungs with a surface area of ≥0.8 m^2^ (Fig. 4, Table 1).

**Table 1:** pH value

Membrane lung	0 ml/min	250 ml/min	500 ml/min	750 ml/min	1000 ml/min
0.4 m^2^	7.06 ± 0.08	7.13 ± 0.08	7.15 ± 0.08	7.17 ± 0.09	7.19 ± 0.08
0.8 m^2^	7.03 ± 0.07	7.11 ± 0.06	7.17 ± 0.05	7.23 ± 0.06	7.27 ± 0.05
1.0 m^2^	7.08 ± 0.03	7.17 ± 0.02	7.24 ± 0.02	7.32 ± 0.03	7.35 ± 0.04
1.3 m^2^	7.01 ± 0.06	7.08 ± 0.07	7.16 ± 0.06	7.23 ± 0.02	7.30 ± 0.05

**Table 2:** PaCO_2_

Membrane lung	0 ml/min	250 ml/min	500 ml/min	750 ml/min	1000 ml/min
0.4 m^2^	111.3 ± 31.5	91.5 ± 22.4	89.9 ± 21.1	86.4 ± 20.6	79.1 ± 18.1
0.8 m^2^	137.3 ± 25.0	107.6 ± 10.2	87.8 ± 8.5	75.7 ± 7.6	68.2 ± 7.7
1.0 m^2^	118.4 ± 19.7	96.6 ± 9.5	81.4 ± 9.4	67.6 ± 10.7	59.2 ± 7.6
1.3 m^2^	139.4 ± 15.0	108.9 ± 13.6	93.6 ± 13.6	77.1 ± 11.3	65.8 ± 9.5

**Table 3:** Normalized extracorporeal CO_2_ elimination

Membrane lung	250 ml/min	500 ml/min	750 ml/min	1000 ml/min
0.4 m^2^	21.2 ± 3.6	33.6 ± 7.7	37.9 ± 5.3	46.5 ± 5.8
0.8 m^2^	27.5 ± 3.7	41.3 ± 6.9	56.9 ± 7.6	69.2 ± 9.2
1.0 m^2^	25.6 ± 2.6	46.5 ± 6.5	62.1 ± 7.4	77.3 ± 4,8
1.3 m^2^	26.5 ± 3.8	50.1 ± 5.0	67.4 ± 4.9	81.1 ± 9.6

**Fig. 5:**
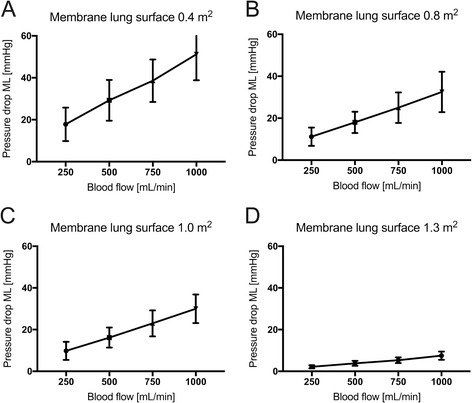
Pressure drop across the membrane lung depending on blood flow. Membrane lung surface ranges from 0.4 m^2^ (**a**), 0.8 m^2^ (**b**), 1.0 m^2^ (**c**) to 1.3 m2 (**d**) with a sweep gas flow of 8 L O_2_/min. Blood flow was titrated from 250 to 1000 ml/min. Each *data point* represents the mean and standard deviation of seven pigs

Oxygen transfer was comparable under all conditions with all membrane lungs independent of the surface area of the membrane lung and linearly dependent on blood flow rates. Maximal oxygen transfer was approximately 60 ml/min, which could be achieved with 1000 ml blood flow/min (Additional file 3: Figure S2). The pressure drop across the membrane lung was determined under each condition. The pressure drop was highest in the 0.4 m^2^ membrane lung: 51.2 ± 12.4 mmHg, with 1000 ml blood flow/min (Fig. 5), and lower across the 0.8 and 1.0 m^2^ surface area membrane lungs (32.5 and 30 mmHg, respectively). Only the largest membrane lung revealed a considerably lower pressure drop of less than 10 mmHg.

The extracorporeal system was most effective with high partial pressures of CO_2_ before the membrane lung. Low blood flow rates of 250 ml/min resulted in a longer blood/gas contact time and demonstrated the lowest partial pressure of CO_2_ post membrane lung (Additional file 1: Figure S1).

## Discussion

The main findings of the present porcine study are that the surface area of the membrane lungs substantially impacts the ability to remove CO_2_ when using vv-ECCO_2_R, and that there is an important interplay between the size of the membrane lung and the chosen blood flow rate. In this regard, we demonstrated that only membrane lungs with a surface area of 0.8 m^2^ or more are capable of fully correcting severe respiratory acidosis, and only when the blood flow is not lower than 1000 mL/min. In turn, smaller membrane lungs are not capable of sufficiently removing CO_2_, even if blood flow rates up to 1000 ml/min are used. For better comparison of the efficiency of the membrane lung, normalized CO_2_ removal was calculated by normalizing the partial pressure of carbon dioxide before the membrane lung to 45 mmHg [28]. With regard to normalization, a surface area of 1.0 m^2^ was optimal to correct severe respiratory acidosis in this experimental porcine model again given that the blood flow is not less than 750 mL/min.

In a previous porcine model study, we have shown that vv-ECCO_2_R was most effective to correct severe respiratory acidosis when using blood flow rates ranging between 750 and 1000 mL/min, while an increase in sweep gas flow from 8 to 16 L/min had no relevant effect on extracorporeal CO_2_ removal [1]. The present study confirms these findings by showing that blood flow rates lower than 750 mL/min are not sufficient to correct severe forms of respiratory acidosis. These animal data are in line with a very recent study by Crotti et al. [29], demonstrating the ability of ECCO_2_R, with 120–160 ml CO_2_ removal/min, to permit spontaneous breathing in acute exacerbations of COPD, without excessively high work of breathing. In addition, the current study adds to these findings as it has shown that an increase of the surface area of the membrane lung is not sufficient to compensate for impaired CO_2_ elimination when low blood flow rates are maintained. In turn, even higher blood flow rates are not capable of fully correcting severe acidosis if small surface area membrane lungs (0.4 m^2^) are used.

Thus, the present study has clearly shown that—from a physiological point of view—the capability of CO_2_ elimination of different systems used for ECCO_2_R is dependent on a complex interplay between the surface area of the membrane lung and the chosen blood flow rate. For sufficiently effective ECCO_2_R, there are minimum requirements for both parameters as outlined above. However, less severe respiratory acidosis can be corrected with low blood flow rates (250–500 ml/min) and a low surface membrane lung (0.4 m^2^). Under these conditions a larger membrane lung has no additional effect and may even worsen some complications of the therapy due to a higher clotting tendency [21, 22].

The present study did not investigate the impact of the sweep gas flow rate, but this has been systematically studied in the previous porcine model study [1]. In that study, CO_2_ elimination was shown to be impaired at constant blood flow rates and oxygenator surface areas, respectively, when using sweep gas flow rates lower than 6 L/min, but CO_2_ elimination could not be substantially increased when using sweep gas flow rates of 8 L/min or higher. This was the reason for choosing a sweep gas flow rate of 8 L/min in the present study.

The current results have some clinical implications. First, the attempt to increase the CO_2_ elimination capability when using ECCO_2_R depends on two important parameters, which are not independent from each other: the blood flow rate and the membrane lung surface area. Importantly, low blood flow rates cannot be entirely compensated for by an increase in the surface area of the membrane oxygenator. The converse is also true: low surface areas cannot be compensated for by high blood flow rates. Second, severe respiratory acidosis can only be sufficiently corrected when using a membrane lung surface area of at least 0.8 m^2^ and blood flow rates of at least 750 mL/min. Third, smaller membrane lung surface areas indeed produced a higher pressure drop across the membrane lung compared to larger surface areas. A lower pressure gradient, however, produces, theoretically, a better blood compatibility and less hemolysis, thus, favoring larger surface areas for clinical application [24]. This advantage of larger membrane lungs, however, must be balanced against the possibility of low blood velocity increasing the incidence of thrombotic deposition, i.e., clotting [22]. Nevertheless, in view of the present results and the current literature, the combination of low blood flow rates and large surface areas should be avoided in clinical practice as the capability of vv-ECCO_2_R under these circumstances is suboptimal, while the risk of clotting is magnified. Finally, we confirmed that oxygenation of the currently tested systems for vv-ECCO_2_R is sparse. Therefore, substantially higher blood flow rates than currently used for vv-ECCO_2_R are clinically reasonable when respiratory acidosis co-exists with significant pulmonary failure also impairing oxygenation, such as in an acute exacerbation of COPD with concomitant pneumonia. In this scenario, there may be a need to transition from vv-ECMO used for pure ECCO_2_R to vv-ECMO set for improving oxygenation as, would be used for a patient with severe ARDS.

The present study has some limitations; most of them are related to the porcine model and the calculated CO_2_ removal. First, data acquired in pigs cannot automatically be transferred into the clinical scenario; however, it has been shown that CO_2_ production in pigs is comparable to CO_2_ production observed in adult humans requiring mechanical ventilation [27]. Therefore, the present data are also likely to be helpful in understanding the physiology of vv-ECCO_2_R in humans. Although, the CO_2_ removal capacity was calculated from CO_2_ content in the blood before and after the membrane lung, instead of direct measurement in the exhaust, the difference is expected to be small. Second, the typical clinical scenario of exacerbated COPD with severe airflow limitation was not simulated, and this limitation has also been outlined in the previous porcine model study [1]. Therefore, the interaction between vv-ECCO_2_R and mechanical ventilation could not be investigated. It is, however, conceivable that removing CO_2_ in a COPD patient with acute respiratory acidosis might be insufficient when using low blood flow rates or small membrane lungs, but the removal of some amount of CO_2_ could positively impact on respiratory drive, work of breathing [30] and respiratory rate, thereby decreasing CO_2_ production that subsequently further offsets respiratory acidosis. Therefore, we must temper our conclusions regarding the clinical effectiveness of different systems used for vv-ECCO_2_R in the clinical setting. In this regard, blood flow rates of considerably lower than 750 mL/min used for vv-ECCO_2_R have been shown to be clinically sufficient to avoid endotracheal intubation in exacerbated COPD patients with acute on chronic hypercapnic respiratory failure presenting without severe respiratory acidosis, who were at risk for NIV failure [2]. This raises the issue of what degree of CO_2_ removal is needed in the clinical setting, as there may be situations when partial CO_2_ removal is effective. This is either because, as above, there is a downstream effect on CO_2_ production, or because normal CO_2_ clearance is not required clinically to exert a positive effect on patient outcomes. Third, even though different surface areas of the membrane lungs were tested, only the rhomboid oxygenator type was investigated, other forms were not. Therefore, the present results are only valid for the rhomboid type of membrane oxygenator, even though the surface area is still thought to be the most important issue characterizing different membrane lungs in terms of their capability to sufficiently remove CO_2_. Last, only one sweep gas flow rate was used during the time course of the experiments, higher flow rates may increase the capacity to remove CO_2_. However, we previously noted, at least with the 1 m^2^ membrane lung, only a small increase in CO_2_ removal with higher sweep gas flow rates [1].

## Conclusions

In conclusion, in this porcine model, vv-ECCO_2_R was most effective when using blood flow rates ranging between 750 and 1000 ml/min, with a membrane lung surface of ≥0.8 m^2^. In contrast, low blood flow rates (250–500 ml/min) were not sufficient to fully correct severe respiratory acidosis, irrespective of the surface area of the membrane lung being used. The converse was also true, low surface membrane lungs (0.4 m^2^) were not capable of completely correcting severe respiratory acidosis across the range of blood flows used in this study.

## Additional files

Additional file 1: Figure S1. Arterial and venous CO_2_ before and after membrane lung under different blood flow conditions (250–1000 ml/min) with different surfaces. Each data point represents the mean and standard deviation of seven pigs. (PDF 6689 kb)

Additional file 2: Figure S3. Extracorporeal elimination of carbon dioxide (CO_2_) depending on membrane lung surface. Extracorporeal CO_2_ elimination was plotted against membrane lung surface. Blood flow was titrated from 250 to 1000 ml/min. Each *data point* represents the mean and standard deviation of seven pigs. (PDF 1291 kb)

Additional file 3: Figure S2. Extracorporeal oxygentransfer depending on blood flow. Membrane lung surface ranges from 0.4 m^2^ (A), 0.8 m^2^ (B), 1.0 m^2^ (C) to 1.3 m2 (D) with a sweep gas flow of 8 L O_2_/min. Blood flow was titrated from 250 to 1000 ml/min. Each data point represents the mean and standard deviation of seven pigs. (PDF 891 kb)

## Abbreviations

ARDS: Acute respiratory distress syndrome
av-ECCO_2_R: Arterio-venous extracorporeal CO_2_ removal
COPD: Chronic obstructive pulmonary disease
ECCO_2_R: Extracorporeal CO_2_ removal
ECMO: Extracorporeal membrane oxygenation
vv-ECCO_2_R: Veno-venous extracorporeal CO_2_ removal
