# Supplementary figures and images for: Impact of membrane lung surface area and blood flow on extracorporeal CO2 removal during severe respiratory acidosis
Source: Intensive Care Med Exp. 2017 Aug 1;5:34. doi: 10.1186/s40635-017-0147-0 (PMC5539069; doi:10.1186/s40635-017-0147-0)

Supplemental Figure 1

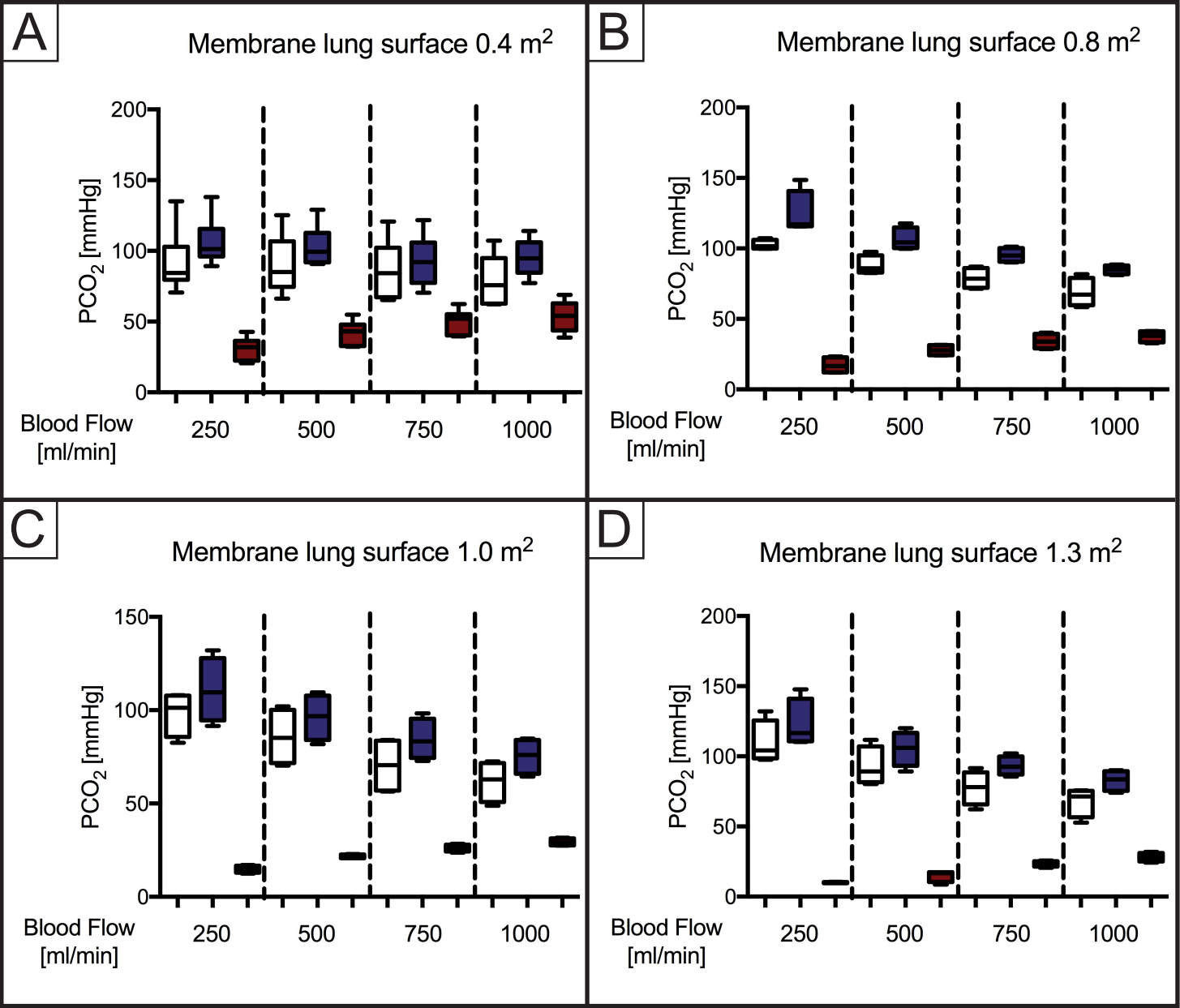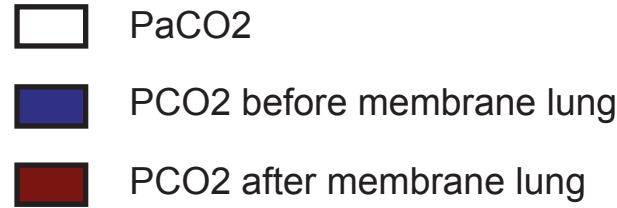

Supplement: Supplementary file 1 — Arterial and venous CO2 before and after membrane lung under different blood flow conditions (250–1000 ml/min) with different surfaces. Each data point represents the mean and standard deviation of seven pigs. (PDF 6689 kb) [file 40635_2017_147_MOESM1_ESM.pdf]

Supplemental Figure 3

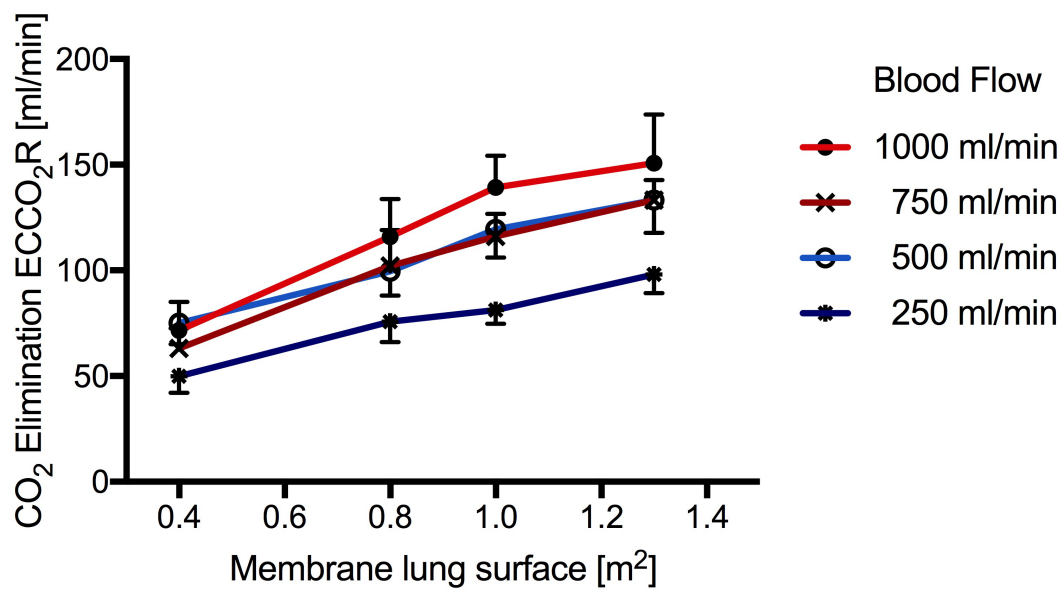

Supplement: Supplementary file 2 — Extracorporeal elimination of carbon dioxide (CO2) depending on membrane lung surface. Extracorporeal CO2 elimination was plotted against membrane lung surface. Blood flow was titrated from 250 to 1000 ml/min. Each data point represents the mean and standard deviation of seven pigs. (PDF 1291 kb) [file 40635_2017_147_MOESM2_ESM.pdf]

Supplemental Figure 2

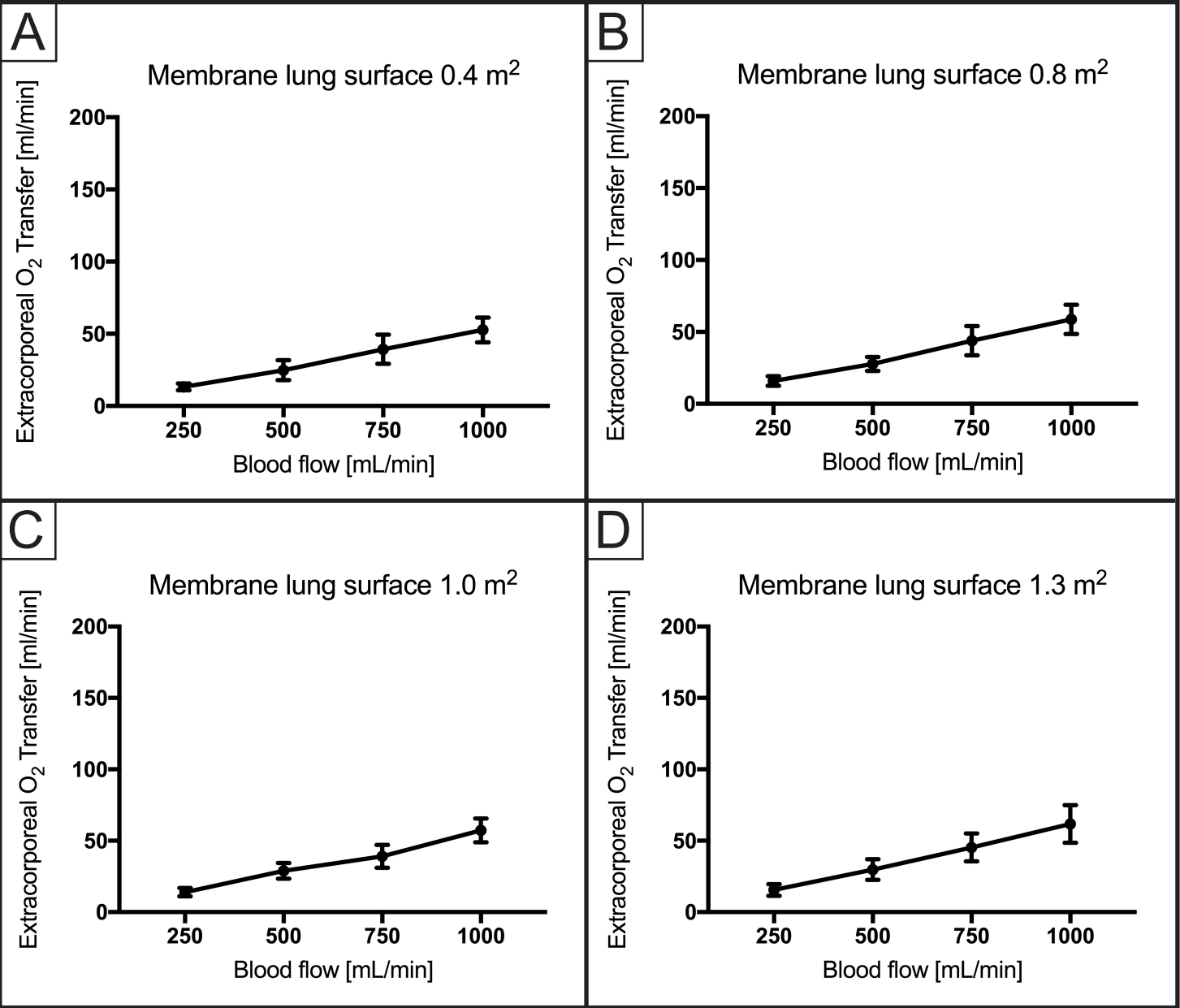

Supplement: Supplementary file 3 — Extracorporeal oxygentransfer depending on blood flow. Membrane lung surface ranges from 0.4 m2 (A), 0.8 m2 (B), 1.0 m2 (C) to 1.3 m2 (D) with a sweep gas flow of 8 L O2/min. Blood flow was titrated from 250 to 1000 ml/min. Each data point represents the mean and standard deviation of seven pigs. (PDF 891 kb) [file 40635_2017_147_MOESM3_ESM.pdf]
